# Knockdown of HCK promotes HREC cell viability and inner blood–retinal barrier integrity by regulating the AMPK signaling pathway

**DOI:** 10.1515/biol-2022-0924

**Published:** 2024-09-03

**Authors:** Lu Chen, Chengmin Lin

**Affiliations:** Ophthalmology Teaching and Research Office, Zhejiang Industry & Trade Vocational College, Wenzhou, Zhejiang, 325000, China; Department of Ophthalmology, Wenzhou Hospital of Integrated Traditional Chinese and Western Medicine, No.75 Jinxiu Road, Wenzhou, Zhejiang, 325000, China

**Keywords:** HCK, DR, HG, internal blood–retinal barrier, AMPK pathway

## Abstract

Diabetic retinopathy (DR), a major complication of diabetes causing blindness, is characterized by retinal damage due to capillary degeneration and vascular leakage. Current treatments are not fully effective, highlighting the need for searching new therapeutic targets. Hematopoietic cell kinase (HCK), a protein involved in various diseases, has been identified as a potential biomarker in DR, but its role in disease progression requires further investigation. Here we investigated the role of HCK in DR and its potential mechanism. We found the expression of HCK increased under the stimulation of high glucose (HG) in human retinal capillary endothelial cells (HRECs). Knockdown of HCK can improve HREC cell viability and the integrity of the internal blood–retinal barrier. HCK depletion suppressed the AMPK pathway in HG-induced HRECs. In summary, HCK may be a potential target for the treatment of DR, which provides a theoretical basis for the development of new treatment strategies.

## 1 Introduction

Diabetic retinopathy (DR) is one of the most common microvascular complications of diabetes, and also one of the main causes of visual impairment and blindness in middle-aged and elderly people [1,2]. DR is often considered a microvascular disease characterized by capillary degeneration, pericyte loss, and vascular leakage [3]. At present, the treatment of DR mainly includes laser photocoagulation, vitrectomy, anti-vascular endothelial growth factor (VEGF) drug therapy, and steroid therapy [4]. These methods have improved the prognosis of patients with DR to some extent, but have not completely prevented the progression of retinal damage [5]. In addition, many patients with DR do not respond well to these treatments, ultimately leading to poor vision outcomes [6]. Therefore, finding new therapeutic targets to mitigate retinal damage is critical to improving clinical outcomes for patients with DR.

Hematopoietic cell kinase (HCK), a member of the Src family of protein tyrosine kinases, is a non-receptor or cytoplasmic tyrosine kinase [7,8]. HCK regulates a variety of cellular processes, including mitosis, differentiation, survival, migration, and adhesion [7,9]. A growing body of research shows that HCK plays a role in a variety of diseases, such as inflammation, fibrosis, and cancer [8,10]. For example, HCK induces macrophage activation by inhibiting autophagy, promoting kidney inflammation, and fibrosis [11]. In glioblastoma, HCK is involved in disease progression by mediating the epithelial–mesenchymal transformation process and may be a potential therapeutic target for glioblastoma [12]. HCK also plays a role in neuronal apoptosis after cerebral hemorrhage and acute myeloid leukemia [12]. Recent studies have found that HCK is one of the immune-related biomarkers in the retina of patients with DR, but its role and mechanism in hyperglycemic-induced retinopathy are still unclear.

The purpose of this study was to investigate the role of HCK in DR and its potential mechanism. Our study found that HCK knockdown can promote the viability of human retinal capillary endothelial cells (HRECs) and the integrity of the internal blood–retinal barrier by regulating AMPK pathway. These findings provide new insights into the role of HCK in DR and provide a theoretical basis for developing therapeutic strategies against HCK. Therefore, we thought HCK could serve as a promising target for DR.

## 2 Materials and methods

### 2.1 Establishment of DR cell model

HRECs were obtained from ATCC (Catalog No. CRL-1780, Manassas, VA, USA) and maintained in DMEM (Gibco, Thermo Fisher Scientific, Waltham, MA, USA) with 10% FBS (Gibco, Thermo Fisher Scientific, Waltham, MA, USA) in a 5% CO_2_ atmosphere. HREC cells were treated with 40 mM glucose (Sigma, St Louis, MO, USA) for 24 h, then the cell model was used as an *in vitro* DR model. High glucose (HG) group: HREC cells were treated with 40 mM glucose (Sigma, St Louis, MO, USA) for 24 h to mimic hyperglycemic conditions observed in DR. Low glucose group: HREC cells were treated with 5.5 mM glucose (Sigma, St Louis, MO, USA), representing normal glucose conditions. Osmotic control group: HREC cells were treated with 5.5 mM glucose plus 34.5 mM mannitol (Sigma, St Louis, MO, USA) to control the osmotic effects of HG concentration. The DR cell model has been constructed according to the previous study [2].

### 2.2 Cell transfection

After 24 h of culture, Lipofectamine^®^3000 reagent (Invitrogen, Carlsbad, CA, USA) was used to transfect si-NC (negative control) and si-HCK (obtained from Riobio, Guangzhou, China), respectively, into the cells. About 5 µL of Lipofectamine^®^ 3000 reagent was diluted in 125 µL of Opti-MEM^®^ medium (Gibco, Thermo Fisher Scientific, Waltham, MA, USA) and incubated for 5 min at room temperature. In a separate tube, 2.5 µg of plasmid DNA or siRNA was diluted in 125 µL of Opti-MEM^®^ medium and then combined with the diluted Lipofectamine^®^ 3000 reagent. The mixture was incubated for 15–20 min at room temperature to allow the formation of complex. The DNA–lipid complex was then added dropwise to each well containing cells in 2 mL of fresh growth medium. After 24 h, the subsequent experiments were conducted. si-HCK sequence: 5′-GACGUUUCACGGAGGAGUAdTdT-3′. Target site on HCK gene: the siRNA targets the coding sequence region of the HCK gene, specifically designed to knock down HCK expression effectively.

### 2.3 CCK-8 assay

HRECs upon the indicated treatment for 24 h were plated into 96-well plates, and treated as indicated. The cells were then cultured with CCK-8 mixture (Dojindo Laboratories, Kumamoto, Japan). Finally, the OD450 was measured with a microplate reader (BD Biosciences, Franklin Lakes, NJ, USA).

### 2.4 Lactate dehydrogenase (LDH) release assay

The LDH releasing degree was measured using the kit from Abcam (ab102526; Abcam, Cambridge, UK). About 50 µL of the collected culture medium was transferred to a new 96-well plate. An equal volume of the LDH reaction mix was added to each well and incubated for 30 min at room temperature in the dark. Absorbance measurement was made at 450 nm using a microplate reader (BD Biosciences, USA) [12].

### 2.5 TEER detection

The electrode was placed in Hank’s Balanced Salt Solution (HBSS) preheated to 37°C for equilibrium for 20 min. Cells were seeded at a density of 1 × 10^5^ cells per well in Transwell inserts (Corning, NY, USA) and cultured until they formed a confluent monolayer. Before measurement, the medium was replaced with fresh preheated HBSS, and 0.5 mL HBSS was added to each well on the apical (AP) side for equilibrium for 20 min. The TEER value was measured using the Millicell-ERS2 voltohmmeter (MilliporeSigma, Burlington, MA, USA) according to the manufacturer’s instructions.

### 2.6 Na-F permeability assay

Na-F (Sigma, St Louis, MO, USA) was dissolved in HBSS to a final concentration of 10 μM and applied to the AP side of the Transwell insert. The basolateral (BL) compartment was filled with 0.6 mL of HBSS. The cells were incubated at 37°C for 1 h. After incubation, 100 μL of the medium from the BL compartment was collected and transferred to a 96-well black plate. The fluorescence intensity was measured using a fluorescence microplate reader (Tecan, Männedorf, Switzerland) at an excitation wavelength of 485 nm and an emission wavelength of 530 nm.

### 2.7 Quantitative polymerase chain reaction (qPCR) assay

Total RNA was extracted from the cells using TRIzol reagent (Invitrogen, USA) according to the manufacturer’s instructions. The extracted RNA was then reverse-transcribed into cDNA using the PrimeScript RT reagent kit (Takara, Japan). qPCR was performed using the SYBR-Green Master Mix (Roche, USA) on a LightCycler 480 Real-Time PCR System (Roche, Switzerland) and respective primers. The PCR conditions were as follows: initial denaturation at 95°C for 5 min, followed by 40 cycles of denaturation at 95°C for 10 s, annealing at 60°C for 20 s, and extension at 72°C for 20 s. The used primers are listed as below: HCK: F: 5′-CCCTGTATGATTACGAGGCCA-3′, R: 5′-CACTCCCCGGATTCCTCTAGG-3; ZO-1: F: 5′-AGGAGAGACACGGAAGAGGA-3′, R: 5′-GTGAGTGGGTTGAGGTAGTG-3′; VE-cadherin: F: 5′-CGAGAGGATGGTGAGGAAGA-3′, R: 5′-GGTTCTTGGGCTTGTCTTCA-3′; and GAPDH: F: AGAAGGCTGGGGCTCATTTG, R: AGGGGCCATCCACAGTCTTC’. The relative gene expression levels were calculated using the 2^−ΔΔCt^ method, with GAPDH serving as the internal control [13].

### 2.8 Immunoblotting

RIPA lysate was added to fully lysate cells to extract protein, which was quantitated by BCA reagent, separated by 10% SDS-PAGE, and further transferred to PVDF membrane. The proteins were blocked with 5% milk, and then the corresponding primary antibodies were added and incubated at 4℃ overnight. Primary antibodies against HCK (ab75839, 1:1,000; Abcam), AKT (ab8805, 1:1,000; Abcam), p-AKT (ab38449, 1:1,000; Abcam), mTOR ab134903, 1:500; Abcam), p-mTOR (ab109268, 1:500; Abcam), AMPK (ab32047, 1:500; Abcam), p-AMPK (ab133448, 1:500; Abcam), GAPDH (ab8245; 1:3,000; Abcam), and then secondary antibodies were incubated for 1 h and photographed after chemiluminescence (Thermo, USA). The method was conducted according to the previous study [14]. The relative protein expression levels were quantified using densitometric analysis.

### 2.9 Statistics

GraphPad Prism 5.0 software was used for all statistical analyses. Both parametric and non-parametric tests were employed depending on the data distribution. Parametric tests, such as Student’s *t*-test, were used for normally distributed data, while non-parametric tests, such as the Mann–Whitney *U*-test, were used for data that did not follow a normal distribution. Experiments were repeated at least three times independently to ensure reproducibility. For a small number of samples, non-parametric statistical data processing was typically used. Data were represented as mean ± SD, and a *p*-value <0.05 was considered statistically significant.

## 3 Results

### 3.1 HCK was highly expressed in HRECs under HG treatment

**Figure 1:**
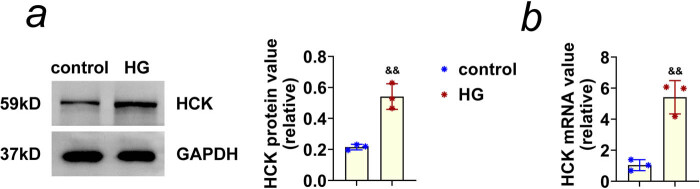
HCK was highly expressed in HRECs under HG treatment. (a) Immunoblot assays showed the expression of HCK in HRECs upon control or the treatment with HG (40 mM glucose) for 24 h. The relative expression of HCK was quantified. (b) qPCR assays showed the mRNA levels of HCK in HRECs upon control or the treatment with HG (40 mM glucose) for 24 h. && *p* < 0.01 vs control. HG, high glucose.

To reveal the possible effects of HCK on DR progression, a DR cell model using HRECs upon treatment of HG (40 mM) for 24 h was first constructed. Through immunoblot assays, we noticed that HG treatment increased the expression of HCK in HRECs (Figure 1a). We further conducted qPCR assays, and the data confirmed that the mRNA levels of HCK in HG-induced HRECs were upregulated (Figure 1b). Therefore, we thought HCK was highly expressed in HRECs under HG treatment.

### 3.2 Knockdown of HCK promoted the viability of HRECs and suppressed the release of LDH

**Figure 2:**
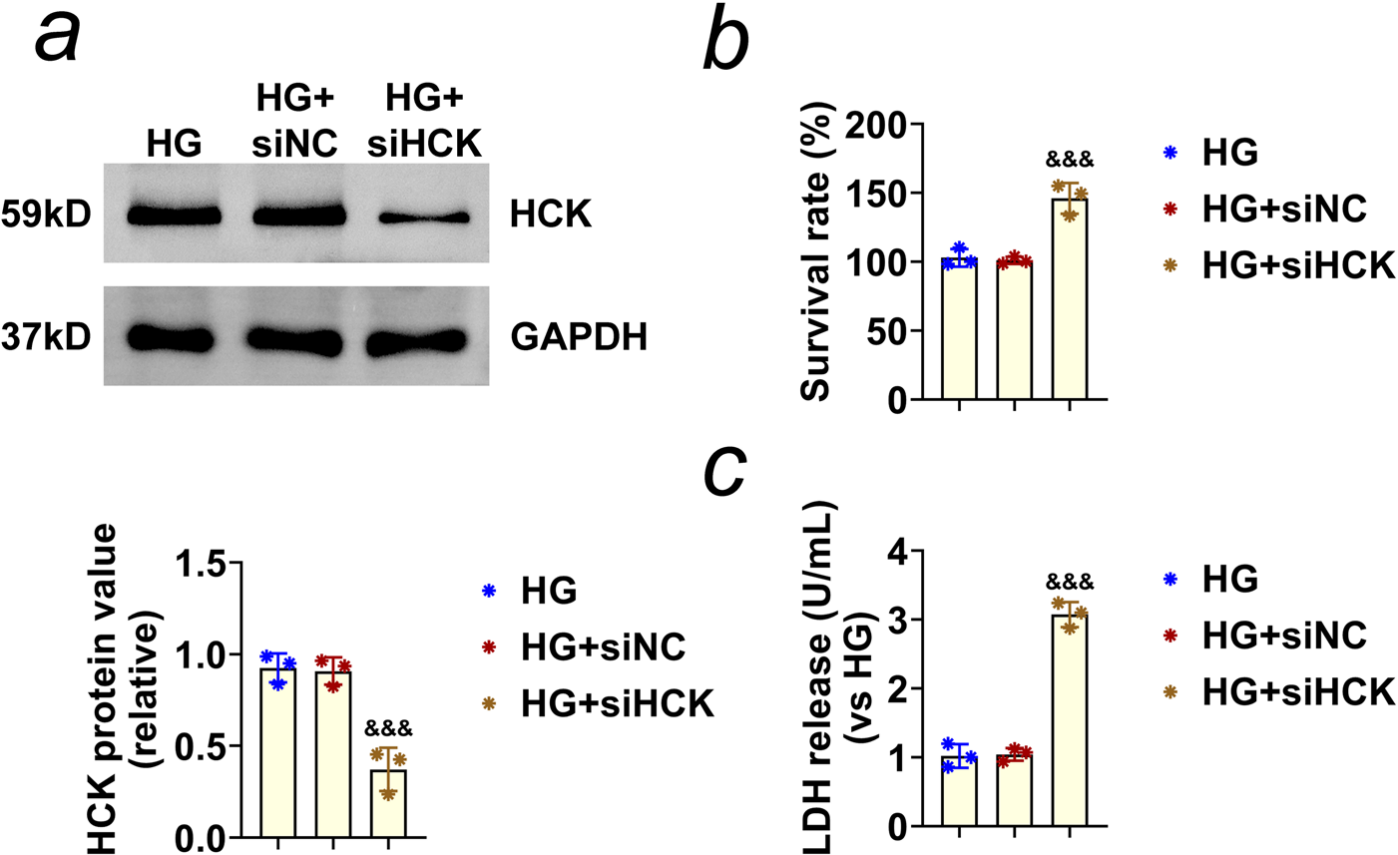
Knockdown of HCK promoted the viability of HRECs and suppressed the release of LDH. (a) Immunoblot assays showed the expression of HCK in HRECs upon treatment with HG (40 mM glucose) and transfection of si-NC or si-HCK for 24 h. (b) CCK-8 assays showed the growth of HRECs upon treatment with HG (40 mM glucose) and transfection of si-NC or si-HCK for 24 h. The OD450 value was measured. (c) LDH detection assays showed the levels of LDH release in HRECs upon treatment with HG (40 mM glucose) and transfection of si-NC or si-HCK for 24 h. &&& *p* < 0.001, si-HCK vs si-NC. HG, high glucose; NC, negative control; LDH, lactate dehydrogenase.

Furthermore, the siRNAs targeting HCK were used and transfected into HRECs to decrease the expression of HCK. Immunoblot assays confirmed the silencing efficiency (Figure 2a). We further conducted CCK-8 assays, and the data showed that HCK depletion promoted the growth of HG-induced HRECs, with the increased OD450 value (Figure 2b). Furthermore, we noticed that knockdown of HCK suppressed LDH levels in HG-induced HRECs through the detection of LDH, suggesting the inhibition of cytotoxicity (Figure 2c). Collectively, these data confirmed that knockdown of HCK promoted the viability of HRECs and suppressed the cytotoxicity.

### 3.3 Knockdown of HCK promotes the integrity of the internal blood–retinal barrier in HRECs upon HG treatment

**Figure 3:**
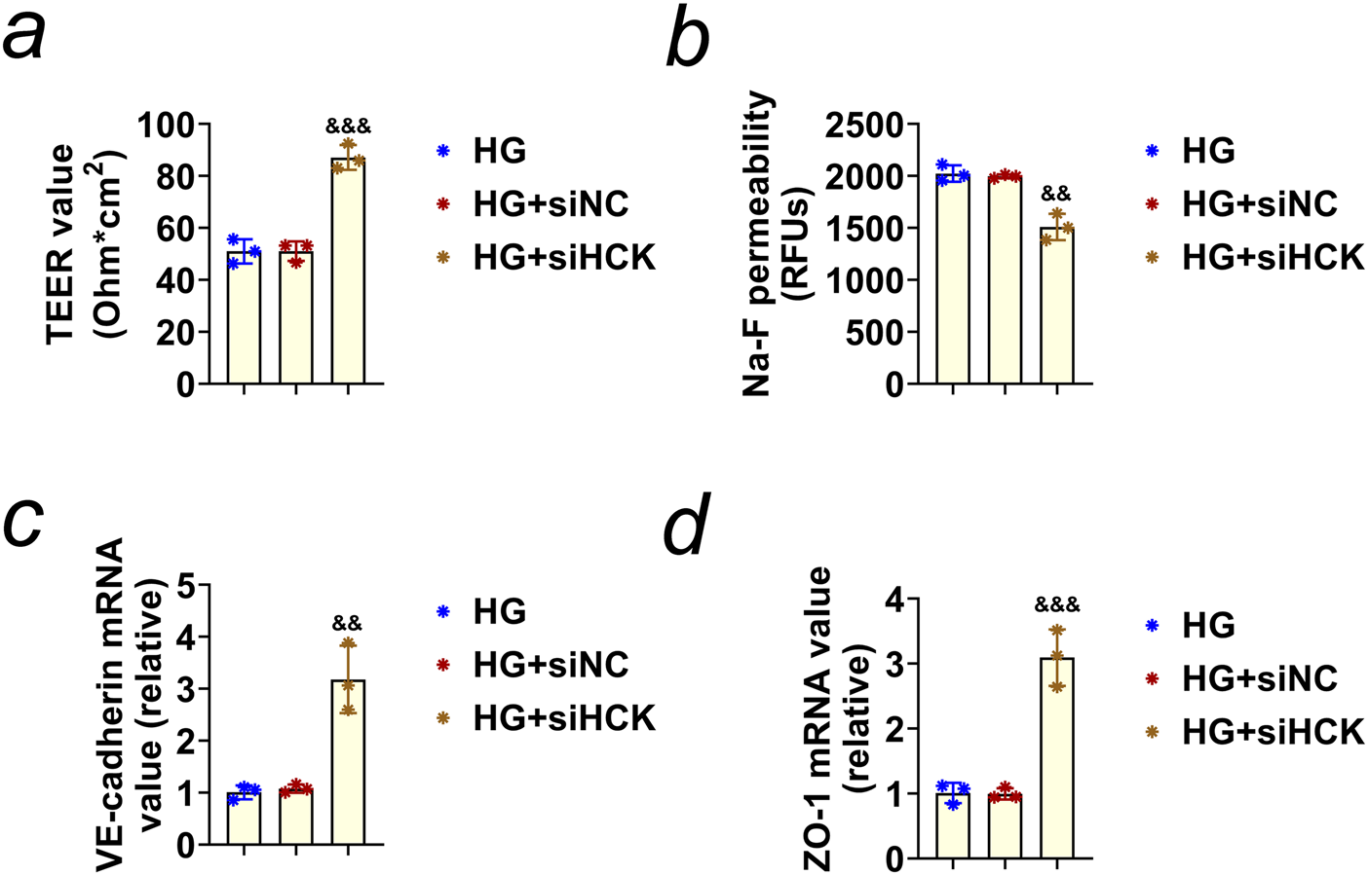
Knockdown of HCK promotes the integrity of the internal blood–retinal barrier. (a) Millicell Resistance System (ERS2) detection showed the TEER value of HRECs upon treatment with HG (40 mM glucose) and transfection of si-NC or si-HCK for 24 h. (b) Na-F permeability of HRECs upon treatment with HG (40 mM glucose) and transfection of si-NC or si-HCK for 24 h. (c) qPCR assays showed the mRNA levels of VE-cadherin in HRECs upon treatment with HG (40 mM glucose) and transfection of si-NC or si-HCK for 24 h. (d) qPCR assays showed the mRNA levels of ZO-1 in HRECs upon treatment with HG (40 mM glucose) and transfection of si-NC or si-HCK for 24 h. && *p* < 0.01, &&& *p* < 0.001, si-HCK vs si-NC. HG, high glucose; NC, negative control.

We then detected the effects of HCK ablation on the integrity of the internal blood–retinal barrier in HG-stimulated HRECs. Through the use of Millicell Resistance System (ERS2), we noticed that depletion of HCK increased the TEER value in HG-stimulated HRECs, suggesting the promotion of the integrity (Figure 3a). Similarly, we noticed that Na-F permeability was decreased in HG-stimulated HRECs, also suggesting the promotion of the integrity (Figure 3b). Consistently, qPCR assays showed that the expression of VE-cadherin and ZO-1, two markers of cell integrity, was increased in HG-stimulated HRECs (Figure 3c and d). Therefore, we thought knockdown of HCK promotes the integrity of the internal blood–retinal barrier in HRECs.

### 3.4 Knockdown of HCK inhibited the AMPK pathway in HG-induced HRECs

**Figure 4:**
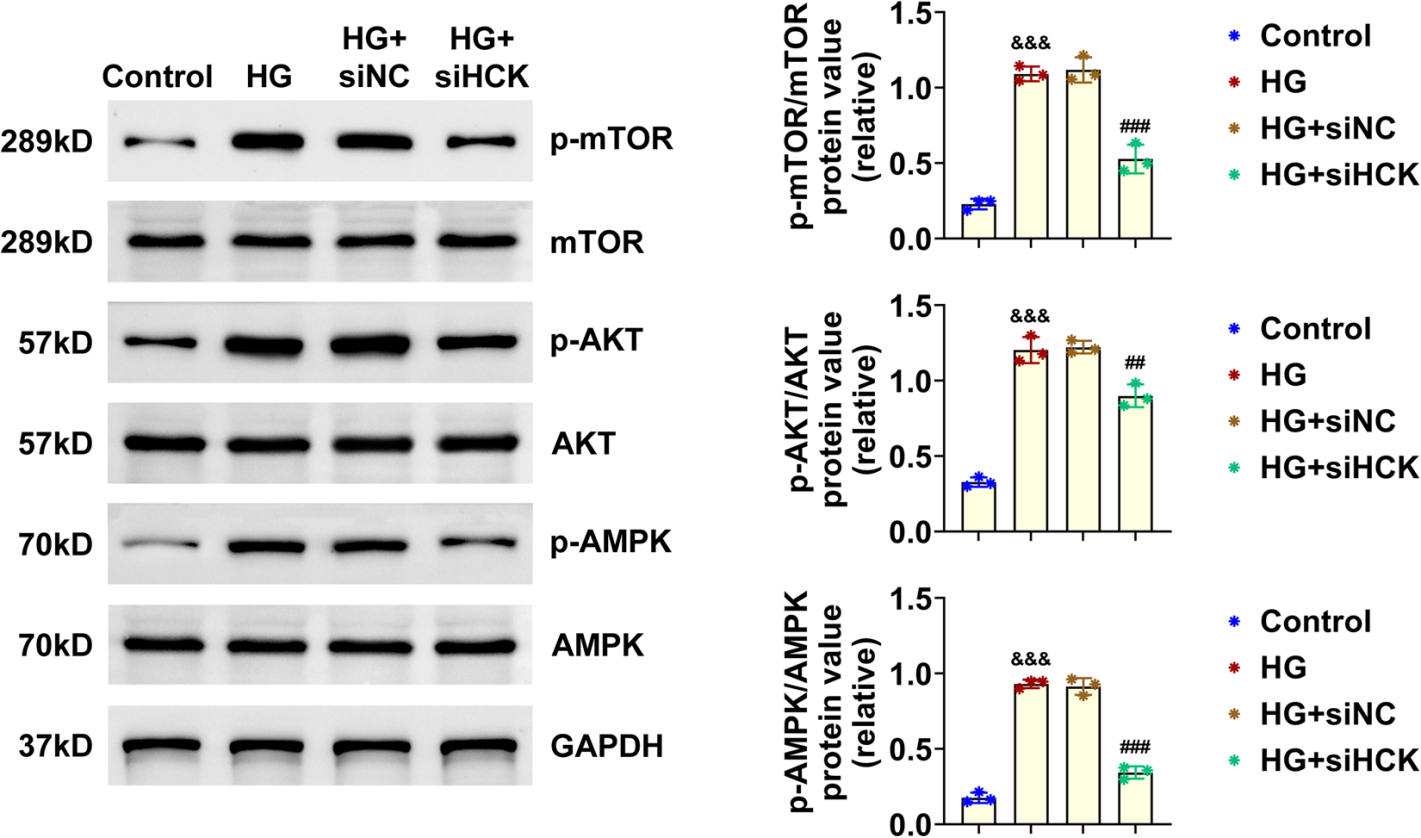
Knockdown of HCK inhibited the AMPK pathway. Immunoblot assays showed the expression and phosphorylation levels of mTOR, AKT, and AMPK in HRECs upon control or treatment with HG (40 mM glucose) and transfection of si-NC or si-HCK for 24 h. The relative phosphorylation levels of mTOR, AKT, and AMPK was quantified. The experiment has been repeated for five times. && *p* < 0.01, &&& *p* < 0.001, si-HCK vs si-NC. HG, high glucose; NC, negative control.

Then, the underlying mechanism was explored through immunoblot assays. AMPK pathway is involved in the regulation of cell growth and integrity, we therefore clarified whether HCK affected these cellular processes of HRECs via AMPK pathway. We noticed that the phosphorylation levels of AKT, mTOR, and AMPK, the three key regulators in AMPK pathway, were increased in HG-induced HRECs, and decreased in HG-induced HRECs after HCK depletion, suggesting the suppression of AMPK pathway (Figure 4). Therefore, knockdown of HCK inhibited the AMPK pathway in HG-induced HRECs.

## 4 Discussion

DR is one of the leading causes of vision loss worldwide. The development of DR is linked to a number of factors, including poor blood sugar control, blood pressure, lipid levels, and the duration of diabetes [6]. The early stage of DR is characterized by structural and functional changes in microvessels, including capillary occlusion, pericyte loss, increased vascular permeability, and microhemangioma formation [2]. As the disease progresses, it may develop proliferative DR, which is characterized by the formation of new blood vessels and retinal or vitreous bleeding [1]. In addition, diabetes-related retinal neuropathy is also an important factor in the development of DR [5]. The current treatment methods for DR mainly include laser photocoagulation, vitrectomy, anti-VEGF therapy, and glucocorticoid therapy [15]. Although these treatments can slow the progression of DR to some extent, they cannot cure DR and may be associated with certain side effects [15]. We found that knockdown of HCK can promote the viability of HRECs and the integrity of the internal blood–retinal barrier by modulating AMPK signaling. This finding provides new insights into the role of HCK in DR and provides a theoretical basis for developing therapeutic strategies through targeting HCK. The obtained findings align with previous clinical studies that have identified HCK as a crucial factor in various inflammatory and fibrotic diseases, underscoring its relevance in DR as well. This highlights the significance of targeting HCK in therapeutic strategies, as it could potentially mitigate retinal damage and improve clinical outcomes of patients with DR.

HCK is a member of the Src family of protein tyrosine kinases that have been shown to play a role in a variety of diseases, including inflammation, fibrosis, and cancer [16,17]. In DR, HCK is considered to be one of the immune-related biomarkers in the retina, but its specific role and the underlying mechanism remains unclear [18]. Our results show that HCK is highly expressed under HG stimulation, which is consistent with previous studies and suggests that HCK may play an important role in the development of DR.

Our findings suggest that HCK plays a significant role in the progression of DR by influencing cell viability and the integrity of the blood–retinal barrier. The increased expression of HCK under HG conditions indicates its potential involvement in the cellular response to hyperglycemia. By knocking down HCK, we observed improved cell viability and reduced cytotoxicity, as evidenced by the decreased release of LDH. This highlights the protective effect of HCK inhibition against HG-induced damage in HRECs. Moreover, the enhancement of the blood–retinal barrier integrity upon HCK knockdown further supports the therapeutic potential of targeting HCK in DR. The increased TEER values and decreased Na-F permeability suggest that HCK depletion strengthens cell junctions, thereby maintaining barrier function. The upregulation of ZO-1 and VE-cadherin also corroborates this protective effect. These results suggest that HCK may play a negative role in DR by affecting cell viability and barrier function. Notably, we also found that knocking down *HCK* can inhibit the expression of AMPK signaling pathway, suggesting that AMPK pathway may be an important pathway for HCK to regulate HREC cell function.

AMPK is a key energy sensor that regulates cell metabolism and survival [19,20]. In DR, activation of the AMPK signaling pathway is thought to be an important mechanism for protecting the retina from HG damage [20,21]. Our findings therefore suggest that HCK may exacerbate the development of DR by inhibiting the AMPK signaling pathway. Further study of this mechanism will help reveal the role of HCK in DR and provide clues for the development of new therapeutic strategies.

Although our study provides new insights into the role of HCK in DR, there are still some limitations. First, our study is primarily based on cell models, and we need to validate our findings in animal models and clinical samples in the future. Second, the specific action mechanism of AMPK pathway and how HCK regulates this pathway still need to be further studied. Additionally, apoptosis was not determined by flow cytometry, which could provide more detailed insights into cell death mechanisms. In addition, exploring the interaction of HCK with other signaling pathways will also help to fully understand its role in DR.

We further found that HCK knockdown can promote HREC cell viability, inhibit LDH release, and promote the integrity of the internal blood–retinal barrier. LDH release is a well-established test for determining total cell necrosis, as it measures the release of LDH enzyme from damaged cells into the culture medium.

Our findings align with previous studies showing that the AMPK pathway plays a crucial protective role in cellular metabolism and survival under hyperglycemic conditions, suggesting that HCK may exacerbate DR progression by negatively regulating this pathway. Furthermore, targeting HCK could offer a novel therapeutic approach to mitigate retinal damage and improve endothelial cell viability, as evidenced by similar protective effects seen with AMPK activation in DR models.

In summary, through the experimental study of HRECs, we found that the expression of HCK increased under the stimulation of HG, which is related to retinal injury. By activating AMPK signaling pathway, HCK knockdown can improve the viability of HREC cells and the integrity of the internal blood–retinal barrier, thereby reducing retinal damage caused by HG. These results suggest that HCK may be a potential target for DR therapy and provide a theoretical basis for developing new therapeutic strategies.
