# Corrigendum to “Knockdown of HCK promotes HREC cell viability and inner blood–retinal barrier integrity by regulating the AMPK signaling pathway”

**DOI:** 10.1515/biol-2025-1282

**Published:** 2026-02-03

**Authors:** Lu Chen, Chengmin Lin

**Affiliations:** Ophthalmology Teaching and Research Office, Zhejiang Industry & Trade Vocational College, Wenzhou, 325000, Zhejiang, China; Department of Ophthalmology, Wenzhou Hospital of Integrated Traditional Chinese and Western Medicine, No.75 Jinxiu Road, Wenzhou, Zhejiang, 325000, China

In the published manuscript, Knockdown of HCK promotes HREC cell viability and inner blood–retinal barrier integrity by regulating the AMPK signaling pathway. *Open Life Sciences*. 2024;19(1): 20220924. https://doi.org/10.1515/biol-2022-0924 the authors found an error in Figure 2C. Specifically, the data trends of the three groups have been reversed.

**Figure 2:**
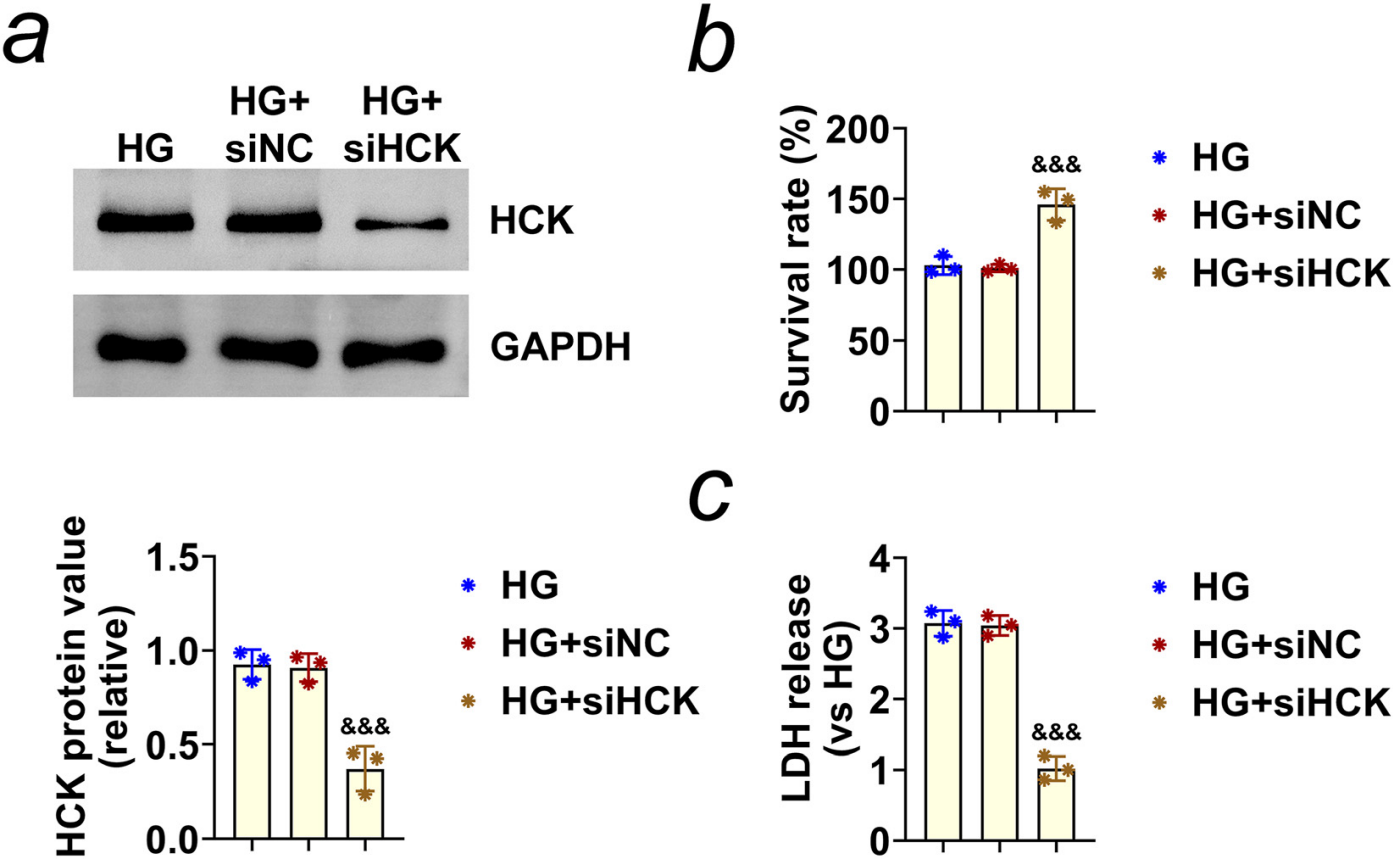


The authors acknowledge the error and state that it was unintentional, unrelated to any academic misconduct, and does not affect the conclusions of the publication. The authors would like to sincerely apologize to the editor, the journal staff, and the readers for the oversight and any inconvenience it may have caused.

Corrected version of Figure 2 is attached below.

